# Effects of glycerol processing on the biomechanical, structural, optical, and antimicrobial properties of human amniotic membrane

**DOI:** 10.1007/s10561-026-10261-z

**Published:** 2026-10-30

**Authors:** Cleopatra Ndhlovu, Adelin Muganza, Lawrence Chauke, Deirdre Kruger

**Affiliations:** 1 Department of Surgery, School of Clinical Medicine, Faculty of Health Sciences, University of the Witwatersrand, Johannesburg, South Africa; 2 Chris Hani Baragwanath Academic Hospital, Johannesburg, South Africa; 3 Department of Obstetrics and Gynaecology, School of Clinical Medicine, Faculty of Health Sciences, University of the Witwatersrand, Johannesburg, South Africa; 4 Charlotte Maxeke Johannesburg Academic Hospital, Johannesburg, South Africa

**Keywords:** Human amniotic membrane, Optical transmittance, Biomaterials processing, Tensile strength, Antimicrobial biomaterials

## Abstract

Human amniotic membrane (hAM) is used as a biological dressing in burn care, and preservation methods may influence its structural and functional characteristics. This study evaluated the effects of a glycerol-based processing and preservation method on properties of hAM. Placentas were obtained from 142 consenting donors following elective caesarean section at a tertiary academic hospital. hAM was processed using ≥99.5% glycerol with agitation cycles and intermediate saline rinses following antibiotic-antifungal decontamination. Paired processed and unprocessed samples from 71 donors were assessed for biomechanical properties, optical transmittance, thickness, bacterial inhibition, and histological characteristics. Statistical analyses included paired t-tests, repeated-measures ANOVA, and effect sizes (Cohen’s d). Processing resulted in modest reductions in ultimate tensile strength (− 7.6%) and tensile stress (− 6.1%), with small effect sizes, while tensile strain was not significantly altered. Optical transmittance increased from 90.8 to 97.1% (p < 0.001; Cohen’s d −0.92), while thickness decreased by 2.9%. Following 12 months of refrigerated storage, glycerol-processed hAM produced larger inhibition zones than unprocessed hAM against all five tested bacterial species under the same conditions. Qualitative histological assessment demonstrated epithelial loss, reduced stromal cellularity, and altered stromal morphology, while the basement membrane remained visibly continuous. Processed hAM demonstrated measurable biomechanical properties and bacterial inhibition, together with increased optical transmittance, and processing-associated histological changes. These in vitro findings characterize the effects of the glycerol-based processing and preservation method but do not establish clinical efficacy. Further in vivo evaluation is required to determine whether these laboratory findings translate into safe and effective clinical performance.

## Introduction

The management of burn wounds aims to restore long term form, function, and quality of life, driving the development of skin substitutes. These materials provide temporary or permanent wound coverage by supporting closure and replacing key functions of the skin, depending on their properties (Farina and Rosique 2013). Compared with synthetic skin substitutes, biological skin substitutes retain a more intact native extracellular matrix structure, which may support the formation of a more natural dermis and promote re-epithelialization (Halim et al. 2010). Biological dressings have demonstrated potential advantages over synthetic dressings in some wound-care settings; for example, collagen-based dressings have been associated with greater epithelialization and reduced pain during dressing changes compared with transparent polyurethane film, although at higher cost (Van den Bergh et al. 2018). Common biological substitutes include cadaveric skin allografts, bovine collagen dressings, porcine xenografts, and human amniotic membrane (hAM). However, their availability and clinical utility are influenced by factors such as donor tissue availability, processing and storage requirements, cost, and the infrastructure required for tissue banking. Cadaveric skin allograft remains an important option for temporary burn wound coverage but is dependent on deceased-donor procurement and established skin-banking infrastructure, which can limit availability, particularly in low- and middle-income countries (LMICs) such as South Africa (Roberson et al. 2020). Xenografts provide an alternative source of temporary biological wound coverage, but limitations including lack of integration, potential rejection, and risk of infection support the continued need for other biological skin substitutes, including human-derived materials (Susini et al. 2025).

In contrast, hAM offers several advantages, including low immunogenicity, natural antimicrobial activity, and the ability to conform closely to wound surfaces. Importantly, it can be obtained from placental tissue that would otherwise be discarded after birth, providing a potentially accessible source of biological material for clinical use (Halim et al. 2010). Although cadaveric skin may be preferred, it remains a limited resource in low and middle income (LMIC) countries like South Africa (Roberson et al. 2020).

In this context, processed hAM may provide a practical and scalable alternative for temporary wound coverage in burn care. However, its potential clinical use depends on processing and preservation methods that maintain relevant structural and functional characteristics while providing adequate storage stability and microbiological safety.

For temporary biological coverage of partial thickness burn wounds, an important function of the dressing is to provide a protective barrier over the wound surface during re-epithelialization.

Preservation of viable donor tissue does not appear to be essential for this role; tissue viability has not been shown to influence the clinical performance of preserved allografts used for partial-thickness burns (Hermans 2011). Accordingly, the present study focused on characteristics relevant to glycerol-preserved hAM as a temporary wound-covering material. A range of preservation strategies has been described, including cryopreservation, lyophilization, dehydration, and glycerol preservation. Published glycerol preservation protocols have employed high glycerol concentrations under refrigerated storage conditions. For example, Maral et al. (1999) preserved hAM in 85% glycerol at 4 °C for more than 1 year, while Zidan et al. (2015) evaluated hAM preserved in 98% glycerol. This approach should be distinguished from cryopreservation protocols in which lower concentrations of glycerol may be used as a cryoprotective agent (CPA) during freezing.

Techniques such as cryopreservation and lyophilization, although effective, require specialized processing and infrastructure including liquid nitrogen tanks, ultra-low freezers or freeze dryers, which may constrain implementation in resource-limited tissue banking environments. These effects are relevant because preservation should ideally maintain the structural characteristics required for handling and biological function while minimizing processing-induced tissue alteration.

Glycerol has been widely investigated as an alternative preservation medium due to its antimicrobial properties and ability to maintain extracellular matrix structure during storage. Despite its frequent use, glycerol-based processing protocols remain poorly standardized with differences in glycerol concentration, temperature, exposure duration, decontamination procedures and adjunct preservation methods potentially contributing to variability in tissue characteristics. Reported outcomes vary with respect to mechanical integrity, structural preservation, optical properties and antimicrobial activity. von Versen-Hoeynck et al. (2008) reported that glycerol-preserved, peracetic acid–ethanol sterilized (PES/GLYC) hAM demonstrated a preservation profile that balanced mechanical robustness with barrier function. However, while peracetic acid is an effective broad spectrum sterilant, its strong oxidative chemistry may contribute to alterations in membrane structure and biomechanical characteristics (von Versen-Hoeynck et al. 2008).

We therefore developed a glycerol-based processing and preservation method for the present study that avoids oxidative sterilants, dehydration, and cryogenic storage. The protocol incorporates antimicrobial agents (0.2% penicillin–streptomycin and 0.05% amphotericin B) with glycerol (60%) and saline (40%) as an initial decontamination step, followed by high purity glycerol processing. We hypothesized that this approach would limit processing-associated alterations while retaining key characteristics of the membrane. The study therefore evaluated the effects of this glycerol-based processing method on the biomechanical, structural, optical, and antimicrobial properties of hAM. Optical transmittance was assessed as a physicochemical characteristic potentially reflecting processing-associated changes in membrane composition and structure, rather than as a primary indicator of clinical performance in burn wound coverage.

## Materials and methods

### Study design

This prospective laboratory based, in vitro experimental study evaluated the effects of a glycerol-based processing method on the properties of hAM. Placentas were obtained from consenting donors recruited at the maternity unit of a tertiary academic hospital and transported under controlled conditions to the research and development laboratory of MagiTech Science (Pty) Ltd, a tissue bank licensed by the South African National Department of Health (License No. J/1/2/4/6 TB-001072025). The facility provided an appropriately controlled environment for the isolation, processing, preservation, and storage of human tissue. Processed hAM was compared with matched unprocessed samples from the same donors to assess processing-associated changes in tensile strength, tensile strain, optical transmittance, thickness, antimicrobial activity, and histological characteristics.

Permission for tissue collection was obtained from the hospital’s Chief Executive Officer and the Head of the Department of Obstetrics and Gynaecology. Ethical approval was granted by the Human Research Ethics Committee (Medical) of the authors’ institution (Clearance certificate no.: M220945), and the study was registered on the National Health Research Database (NHRD) (Ref no.: GP_202111_058). Donor recruitment and screening were conducted in accordance with the requirements of the National Health Act of South Africa (61 of 2003) and its regulations relating to tissue banks (Government Gazette 2011). The study was conducted in accordance with the ethical principles of the Declaration of Helsinki for medical research involving human participants, including research involving identifiable human material and data (World Medical Association 2013).

### Donor recruitment and sample size

Placenta donor recruitment was conducted at a tertiary hospital in the Gauteng Province of South Africa. The hospital provides numerous specialist services including the Department of Obstetrics and Gynaecology, which provides antenatal care services to ‘walk-in’ patients and high-risk patients for whom basic antenatal care is inadequate at the surrounding healthcare centres (Nana et al. 2021). hAM processing and laboratory procedures were performed by the principal investigator within the licensed tissue-banking facility.

Written informed consent was obtained from pregnant women aged ≥18 years with gestational age ≥36 weeks who were scheduled for an elective caesarean section at 38 weeks. Medical history was reviewed from patient records, and maternal blood samples were collected for screening for human immunodeficiency virus types 1 and 2 (HIV-1/2), hepatitis B virus, hepatitis C virus and syphilis.

Exclusion criteria were applied to support tissue safety, sterility, and biological integrity. These included premature rupture of membranes due to the increased risk of ascending infection (Dayal et al. 2024); significant local genital tract infections that could compromise tissue sterility; amniotic infection syndrome as it can affect membrane integrity and lead to microbial contamination of the placenta (Romero et al. 2021); known congenital malformations of the foetus or neonate as they could be associated with underlying infectious or genetic conditions; toxaemia (pre-eclampsia/eclampsia) because of its potential to impair placental function; meconium-stained amniotic fluid as it indicates possible intrauterine distress or inflammation (Gallo et al. 2023); and positive maternal serology for transmissible infections, including HIV, HBV, HCV, or syphilis, due to the risk of disease transmission (Government Gazette 2011).

The sample size calculation was based on the estimated source population of pregnant women aged ≥18 years attending the study hospital maternity ward who were eligible for caesarean section. Hospital records indicated that approximately 350 caesarean sections were performed per month in 2021 (personal communication, March 2022). Based on an estimated source population of approximately 2100 women over the originally planned 6-month recruitment period, the required sample size was calculated using the Raosoft® sample size calculator (Raosoft Inc., Seattle, WA, USA), assuming a 90% confidence level and 5% margin of error. This yielded a target recruitment of 240 women for donor screening. Thirty-nine women declined donation, leaving 201 women available for screening. Following application of eligibility criteria, 59 women were excluded, primarily due to positive HIV serology, resulting in a final cohort of 142 eligible consenting donors. The observed exclusion rate was consistent with the HIV prevalence of 28.6% found in a hospital maternity ward study previously reported (Benali et al. 2019).

### Preparation, processing, and preservation

Upon delivery of the infant in the operating theatre, the placenta was packaged under sterile theatre conditions and transported to the tissue bank laboratory in sterile 0.9% saline solution on wet ice at 2–8 °C. Following receipt, all tissue isolation and processing procedures were performed aseptically within a Class II biological safety cabinet located in the clean room facility of the licensed tissue bank. All tissue manipulation was performed using sterile instruments, sterile consumables, and appropriate personal protective equipment to minimize the risk of exogenous contamination. The amniotic membrane was separated from the placenta by blunt finger dissection. The hAM was manually separated from the chorion using sterile forceps, inspected for residual blood clots and adherent tissue, and repeatedly rinsed with sterile 0.9% saline until no visible blood remained. Prior to processing, matched unprocessed control samples were removed for microbiological, tensile, thickness, and optical transmittance testing. Throughout isolation and subsequent processing, hAM was handled aseptically using sterile instruments and transferred only into sterile containers containing sterile processing solutions.

The glycerol-based hAM processing and preservation method evaluated in this study was developed for the present investigation. High-concentration glycerol preservation of hAM has previously been described, including preservation in 85% glycerol at 4 °C (Maral et al. 1999) and 98% glycerol (Zidan et al. 2015); however, the specific processing sequence described below was developed for this study. Following aseptic isolation and washing, hAM was completely immersed in a 1 L decontamination solution comprising 60% glycerol (Molecular Biology grade, ≥99.5%; Sigma-Aldrich), 40% sterile 0.9% saline, 2 mL Penicillin–Streptomycin (10,000 U/mL penicillin and 10,000 µg/mL streptomycin; Gibco, Thermo Fisher Scientific, Cat. No. 15140122), and 0.5 mL Amphotericin B (250 µg/mL; Gibco, Thermo Fisher Scientific, Cat. No. 15290026). This produced final antimicrobial concentrations of 20 U/mL penicillin, 20 µg/mL streptomycin, and 0.125 µg/mL amphotericin B. The hAM was agitated on a rotary shaker at 100 rpm for 3 h at approximately 23 °C to facilitate uniform exposure to the decontamination medium.

Following the 3-h decontamination step, the membrane was transferred to sterile molecular biology-grade glycerol (≥ 99.5%; Sigma-Aldrich) and agitated at 100 rpm in a shaking incubator set at 37 °C for 3 h. This high-purity glycerol treatment was repeated for three consecutive cycles, with the membrane rinsed in sterile 0.9% saline solution between successive glycerol baths. Following the third cycle, the membrane was measured and transferred to a fresh batch of sterile ≥99.5% molecular-biology-grade glycerol for preservation and storage at 1–4 °C. The protocol therefore comprised an initial glycerol-based antibiotic-antifungal decontamination step followed by three sequential high-purity glycerol treatments with intermediate saline rinsing. Matched unprocessed and processed hAM samples were subsequently evaluated for biomechanical properties, thickness, optical transmittance, antimicrobial inhibition, and histological characteristics. For all comparative assessments reported in this study, unprocessed hAM was maintained in sterile 0.9% saline at 1–4 °C for 12 months and served as the time-matched comparator to glycerol-processed hAM preserved for the same duration and under the same temperature conditions.

### Biomechanical characterization

A pre-specified subset of 71 placentas (50% of the donor cohort) was randomly selected for paired analysis. Since each processed membrane was compared with its matched unprocessed counterpart from the same donor, the paired study design reduced inter-donor variability and improved statistical efficiency. A simple random selection using the GIGAcalculator Random Team Generator (Georgiev n.d.) was performed to preserve the within-donor design and minimize inter-donor variability. This strategy was chosen a priori to ensure feasibility with available instruments; avoid batch effects by spreading testing across the recruitment period; and maintain statistical power for paired comparisons while conserving tissue for microbiology and future validation.

The tests were conducted according to the ASTM D882-12 standard for thin plastic sheeting, adapted for biological soft tissue (ASTM International 2012; D20 Committee 2012). Although adapted from ASTM D882-12, it should be noted that no universally standardized protocol currently exists for tensile testing of biological soft tissues.

Samples were cut to standardized dimensions (30 mm × 25 mm) and allowed to air-dry overnight at room temperature within the controlled laboratory environment, without the use of active drying equipment. This step was performed to reduce membrane slippage during clamping and tensile testing. No active drying equipment or heat was applied. The same preparation and drying procedure was applied to both unprocessed and processed hAM specimens to ensure consistent testing conditions between groups. Tensile properties tests were performed using an Instron 5966 universal testing machine (Instron, Norwood, MA, USA) equipped with a 10 kilonewtons (kN) load cell under ambient laboratory conditions around 23 °C. A nominal specimen thickness of 0.02 mm was used for calculation of tensile stress. Each specimen was secured between the testing grips and subjected to uniaxial tensile loading at a crosshead speed of 20 mm/min until failure. This configuration has been previously validated as appropriate for soft tissues and biomaterials testing (Instron® 2023). Tensile properties tests included Maximum Tensile Strain (MTS), Tensile Stress at Maximum Strength (TSMS), and Ultimate Tensile Strength (UTS).

### Optical characterization

Optical transmittance was also assessed on a subset of 71 placentas (50% of the donor cohort). Random selection was once again applied (Georgiev n.d.). Each placenta provided two matched specimens (processed and unprocessed) to allow for a paired comparison. Optical transmittance was measured using a Perkin Elmer Lambda 750 s UV–Vis spectrophotometer (PerkinElmer Inc., Waltham, MA, USA) across the visible spectrum (400–700 nm) at 1 nm intervals. This wavelength range was selected as it corresponds to clinically relevant visible light transmission through the membrane, enabling bedside inspection of the wound bed, since hAM has been shown to transmit over 70% of light above 400 nm and more than 90% above 600 nm (Ijiri et al. 2006).

### Thickness test

Thickness was assessed for all 142 hAM samples collected in the study to capture inter-donor variability and determine whether processing resulted in a measurable change in membrane thickness. Each membrane was placed between two glass slides, and the combined thickness was measured with and without the membrane using a calibrated digital caliper. Each sample was measured independently by two operators using the slide-subtraction method, and the average of the two readings was used for analysis. This indirect approach was used because the thin and hydrated nature of hAM made reproducible direct measurement with a digital caliper difficult. The use of the full donor set allowed processing-associated differences in thickness to be evaluated across the entire study cohort.

### Antimicrobial activity assay

Antimicrobial activity was assessed in both unprocessed and glycerol-processed hAM, following 12 month of refrigerated storage, against five clinically relevant burn wound pathogens: *Escherichia coli, Klebsiella pneumoniae, Streptococcus pyogenes, Pseudomonas aeruginosa,* and *Staphylococcus aureus* (Mundhada et al. 2015)*.* This comparison was performed to evaluate differences in antimicrobial activity between unprocessed and processed membranes. Prior to antimicrobial testing, glycerol-preserved hAM samples were thoroughly rinsed with sterile 0.9% saline to remove residual glycerol before application to the inoculated culture media. Unprocessed hAM samples were maintained in sterile 0.9% saline and refrigerated for the same duration, then underwent the same antimicrobial testing and served as the comparator.

Freeze-dried reference strains were reconstituted in sterile 0.9% saline and subsequently transferred into nutrient broth for recovery and propagation. Nutrient broth, a liquid, non-selective general purpose medium commonly used for the cultivation and enrichment of non-fastidious bacteria, was employed to ensure optimal bacterial growth prior to testing (Forbes et al. 2007). Bacterial suspensions were adjusted to a 0.5 McFarland turbidity standard (approximately 1–2 × 10^8^ CFU/mL) in accordance with Clinical and Laboratory Standards Institute (CLSI) guidelines for antimicrobial susceptibility testing (Clinical and Laboratory Standards Institute 2023).

Two parallel experiments were conducted. In the first, bacteria were cultured on organism specific media appropriate for their growth characteristics. *S. aureus* on Blood Agar, *K. pneumoniae*, *E. coli*, and *P. aeruginosa* on MacConkey Agar, and *S. pyogenes* on Mueller–Hinton Agar, the CLSI-recommended medium for susceptibility testing (MacFaddin 2000). In the second, the same species were plated on non-selective Nutrient Agar (NA) to provide a baseline evaluation of antimicrobial activity independent of media interactions. In both assays, processed hAM discs were applied using the standard disk diffusion method (European Committee on Antimicrobial Susceptibility Testing (EUCAST 2019) and plates were incubated aerobically at 37 °C for 48 h.

Following incubation, plates were examined for zones of inhibition. For unprocessed hAM, where inhibition zones were small and less readily visible, a colony counter was used to aid visualization and confirm the presence of inhibition zones. Digital photographs were obtained for both unprocessed and glycerol-processed samples, and inhibition-zone diameters were measured from the images in millimeters (mm) using AntibiogramJ software (Alonso et al. 2017). The same image-based measurement method was applied to both groups to ensure consistent quantification. Larger inhibition-zone diameters were interpreted as greater bacterial inhibition under the assay conditions.

### Histological evaluation

Histological evaluation was conducted to assess the preservation of membrane architecture following glycerol processing. Representative samples of both unprocessed and glycerol-processed hAM were fixed in 10% neutral buffered formalin, embedded in paraffin, and sectioned at a thickness of 5 µm. The sections were stained using Hematoxylin and Eosin (H&E) to evaluate overall tissue morphology and cellular structure, Masson’s trichrome to evaluate stromal collagen architecture, and Periodic acid Schiff (PAS) staining to assess basement membrane continuity (Rodríguez‐Ares et al. 2009; Suvarna et al. 2019). All stained sections were examined by light microscopy to compare epithelial morphology and cellularity, stromal architecture, collagen organization and basement membrane continuity between processed and unprocessed membranes.

### Statistical analyses

Statistical analyses were conducted in Python (Jupyter Notebook environment), with data processing conducted using Pandas (McKinney 2010) and analyses implemented using Pingouin library (Vallat 2018). All methods and interpretations were reviewed in consultation with a biostatistician.

The percentage reduction in tensile strength was calculated by comparing processed and unprocessed samples. Paired t-tests were applied to assess differences in tensile strength, tensile stress, and tensile strain, with significance set at p < 0.05. Effect sizes (Cohen’s d) were also calculated to interpret the practical significance of findings.

Transparency test results were analyzed using paired t-tests to evaluate differences between groups, and Cohen’s d was calculated to assess effect size and practical significance.

In terms of thickness testing, paired comparisons were made between processed and unprocessed samples from the same donor. Statistical significance was evaluated using paired t-tests, and effect size (Cohen’s d) was calculated to assess practical relevance, independent of sample size.

For the antimicrobial assays, inhibition-zone diameters are reported as means and standard deviations (SDs). Within each experimental condition, inhibition zone diameters were analyzed using repeated-measures ANOVA with bacterial species as the within-subject factor, thereby controlling for donor-level variability when the same hAM specimen was tested across all species (Kan et al. 2024). Where significant, Tukey-adjusted post-hoc comparisons were performed to identify pairwise differences between species.

Inhibition-zone diameters for the comparison of unprocessed and processed hAM tested on the same organism-specific media, were analyzed using paired statistical tests because samples were matched within donors. For glycerol-processed hAM evaluated under organism-specific versus Nutrient Agar conditions, differences in inhibition-zone diameter were also analyzed using paired test model. Statistical significance was defined as *p* < 0.05.

## Results

### Biomechanical properties test

As shown in Table 1, glycerol processing resulted in modest reductions in tensile strength, tensile stress, and thickness, together with increased in optical transmittance, while maximum tensile strain was not significantly altered.

**Table 1:** Summary of functional properties of unprocessed (Group A) vs processed (Group B) hAM

Parameter	Group A	Group B	% Change	p-value	Cohen’s d
*Biomechanical properties*					
UTS, mean (± SD) MPa	6.6 ± 1.1	6.1 ± 1.2	↓ 7.6	<0.001	0.24
TSMS, mean (± SD) MPa	6.51 ± 1.1	6.11 ± 1.0	↓ 6.1	<0.001	0.20
MTS (%)	8.2 ± 1.3	7.9 ± 1.9	↓ 3.7	0.262	0.15
*Transparency and thickness properties*					
Transparency, % (± SD) transmittance (400–700 nm)	90.82 ± 4.39	97.07 ± 5.81	↑ 6.9	<0.001	0.92
Thickness, mm (± SD)	0.035 ± 0.002	0.034 ± 0.002	↓ 2.9	0.001	0.50

UTS, Ultimate Tensile Strength; TSMS, Tensile Stress at Maximum Strength; MTS, Maximum Tensile Strain; MPa, megapascals

#### Ultimate tensile strength (UTS)

Mean UTS decreased from 6.6 ± 1.1 MPa in unprocessed hAM to 6.1 ± 1.2 MPa following processing, corresponding to a 7.6% reduction (p < 0.001; Cohen’s d = 0.24). Although statistically significant, the magnitude of the difference and effect size were small.

#### Tensile stress

Mean tensile stress at maximum load decreased from 6.51 ± 1.1 MPa in unprocessed hAM to 6.11 ± 1.00 MPa following processing, corresponding to a 6.1% reduction (p < 0.001); Cohen’s *d* = 0.20).

#### Maximum tensile strain (MTS)

Mean MTS decreased from 8.2 ± 1.3% in unprocessed hAM to 7.9 ± 1.9% following processing, corresponding to a 3.7% reduction. This difference was not statistically significant (p = 0.262; Cohen’s *d* = 0.15).

### Optical transmittance

Mean optical transmittance increased from 90.82 ± 4.39% in unprocessed hAM to 97.07 ± 5.81% following glycerol processing, representing an absolute increase of 6.25 percentage points and a relative increase of 6.9% (p < 0.001; Cohen’s d = 0.92).

### Thickness

Mean membrane thickness decreased from 0.035 ± 0.002 mm in unprocessed hAM to 0.034 ± 0.002 mm following processing (p = 0.001; Cohen’s d = 0.50).

### Antimicrobial activity assay

**Fig. 1:**
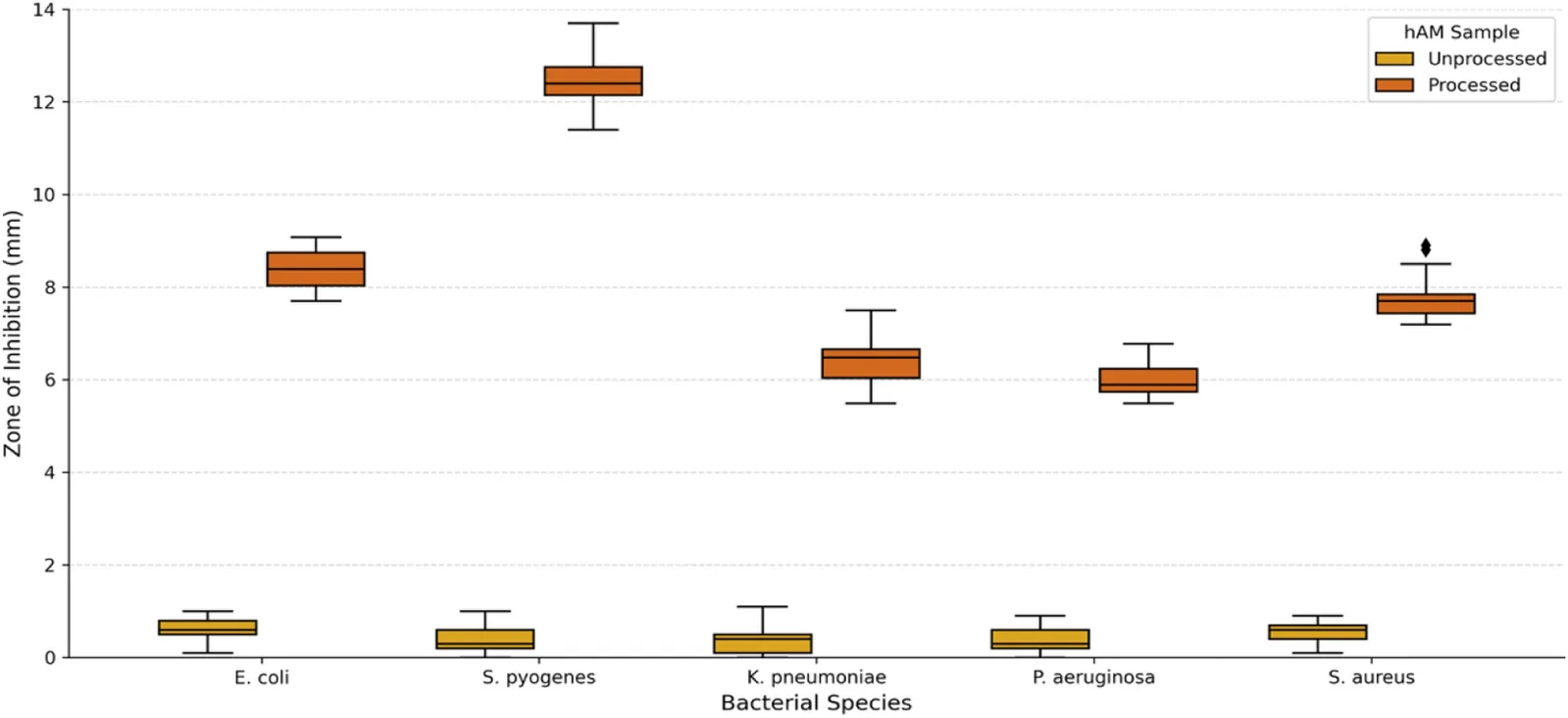
Box plot of inhibition-zone diameters (mm) across processed and unprocessed hAM samples under different media conditions. *E. coli* on MacConkey Agar*, S. pyogenes* on Mueller–Hinton Agar *K. pneumoniae,* and *P. aeruginosa* on MacConkey Agar; and *S. aureus* on Blood Agar. Box plots show the distribution of samples

Figure 1 shows the distribution of inhibition-zone diameters for unprocessed and processed hAM following 12 months of refrigerated storage, with both groups tested on the same organism-specific media. Unprocessed hAM produced small inhibition zones across all five bacterial species, whereas glycerol-processed hAM produced substantially larger inhibition zones under the same assay conditions. This difference was consistently observed across all five bacterial species. Variation in inhibition-zone diameter was observed between samples within each group.

When cultured on organism specific media, processed hAM demonstrated measurable inhibition against all five bacterial species. Inhibition zones differed significantly across species (repeated-measures ANOVA, p < 0.0001). The largest inhibition zones were observed for *Streptococcus pyogenes* (12.42 ± 0.48 mm), followed by *Escherichia coli* (8.37 ± 0.43 mm) and *Staphylococcus aureus* (7.74 ± 0.39 mm). Smaller but consistent inhibition zones were observed for *Klebsiella pneumoniae* (6.44 ± 0.46 mm) and *Pseudomonas aeruginosa* (6.03 ± 0.39 mm). Post-hoc Tukey testing identified significant pairwise differences (p < 0.001). No bacterial growth was detected beneath the hAM discs.

In contrast, unprocessed hAM produced small inhibition zones when tested under the same organism specific conditions. Mean inhibition-zone diameters were 0.61 ± 0.24 mm for *E. coli*, 0.54 ± 0.23 mm for *S. aureus*, 0.41± 0.30 mm for *S.pyogenes*, 0.41 ± 0.30 mm for *K. pneumoniae,* and 0.40 ± 0.27 mm for *P. aeruginosa*. Despite the small absolute magnitude of inhibition, inhibition-zone diameter differed significantly across species (repeated measures ANOVA, F(4,280) = 12.05, p < 0.0001). Tukey-adjusted post-hoc comparisons showed that *E.coli* produced significantly larger inhibition zones than *S. pyogenes*, *K. pneumoniae*, and *P. aeruginosa* (p ≤ 0.0001 for all comparisons), while *S. aureus* also produced significantly larger zones than these three species (p = 0.026, p = 0.020, and p = 0.011, respectively). *E.coli* and *S. aureus* did not differ significantly from one another (p = 0.563), nor did *S. pyogenes*, *K. pneumoniae*, and *P. aeruginosa* differ significantly from one another (p ≥ 0.999).

**Fig. 2:**
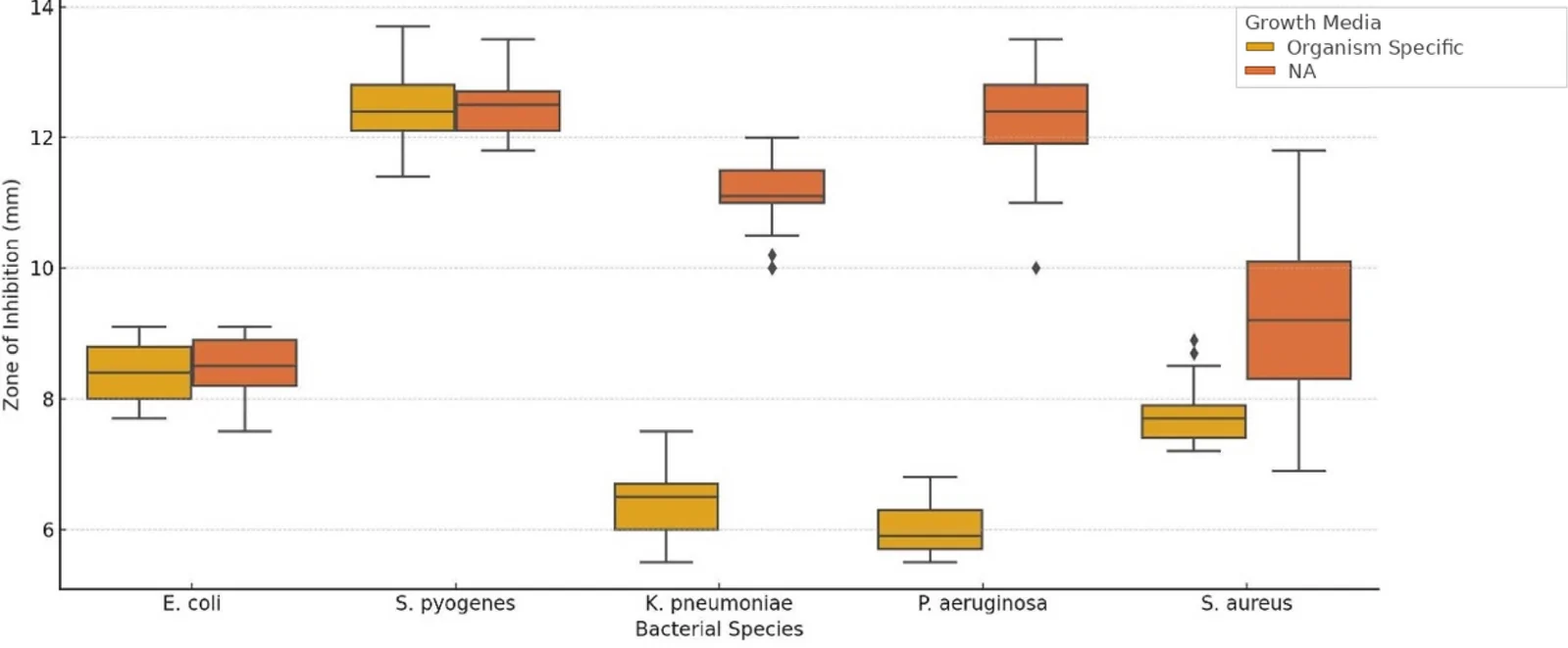
Side by side box plot of processed hAM samples under organism specific and NA (Nutrient Agar) conditions. *Organism specific Agar*= *McConkey, Blood Agar & Mueller Hinton*

Figure 2 shows the distribution of inhibition-zone diameters produced by glycerol-processed hAM against the five bacterial species under organism-specific and Nutrient Agar conditions. Inhibition-zone diameters varied according to bacterial species and culture medium and were generally larger on Nutrient Agar. *S. pyogenes* demonstrated the largest inhibition zones across both media conditions. Inhibition-zone diameters for *E. coli* were similar between the two media conditions, whereas larger differences between media conditions were observed for *P. aeruginosa, K. pneumoniae,* and *S. aureus*. Variation in inhibition-zone diameter was also observed between individual hAM samples.

**Fig. 3:**
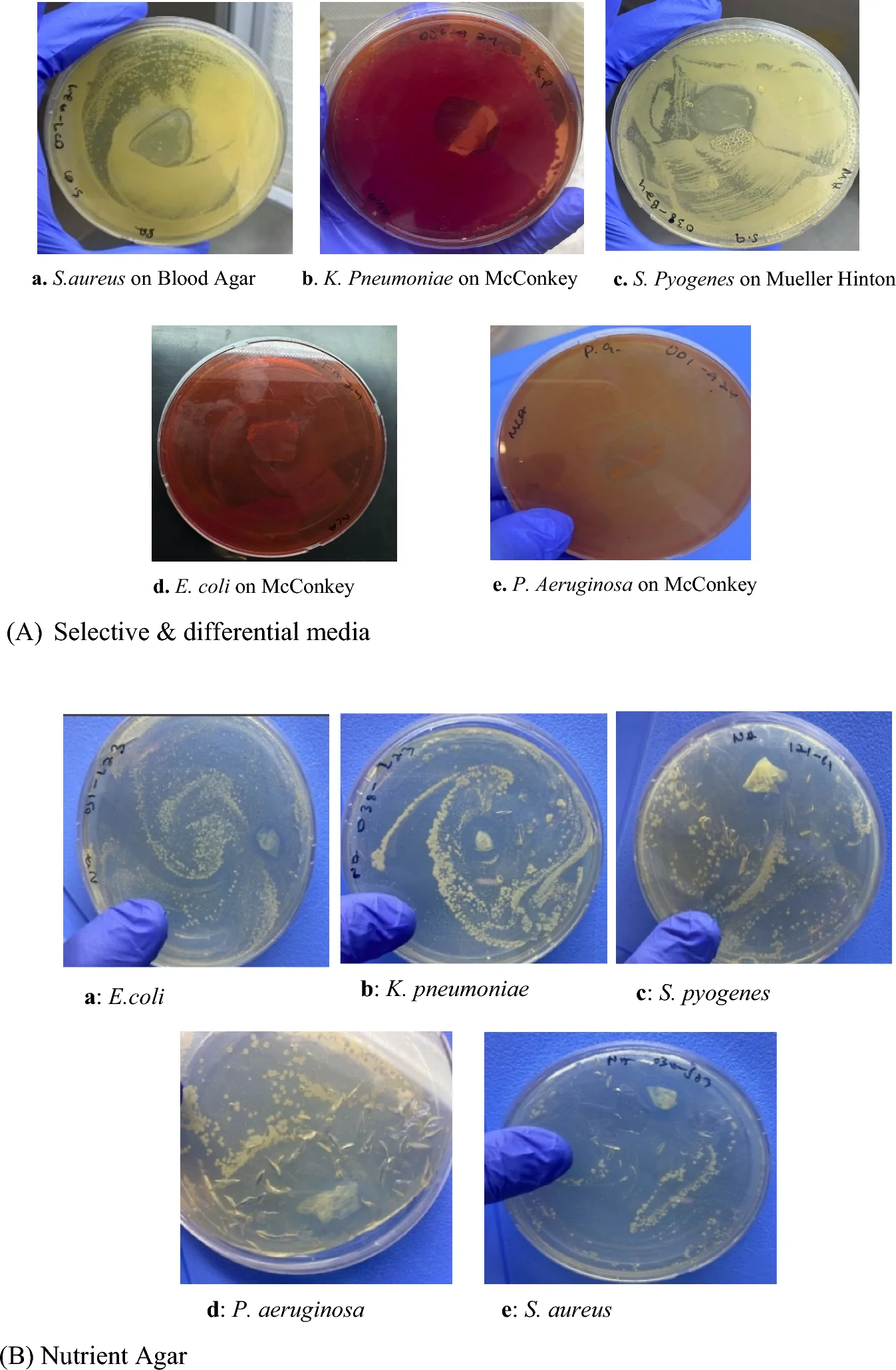
**a** and **b** Antimicrobial activity of glycerol-preserved hAM after 12 months of storage

Under organism-specific media conditions (Fig. 3a), inhibition zones were generally smaller than those observed on nutrient agar (Fig. 3b), although the magnitude of this difference varied among bacterial species.

### Histological evaluation

**Fig. 4:**
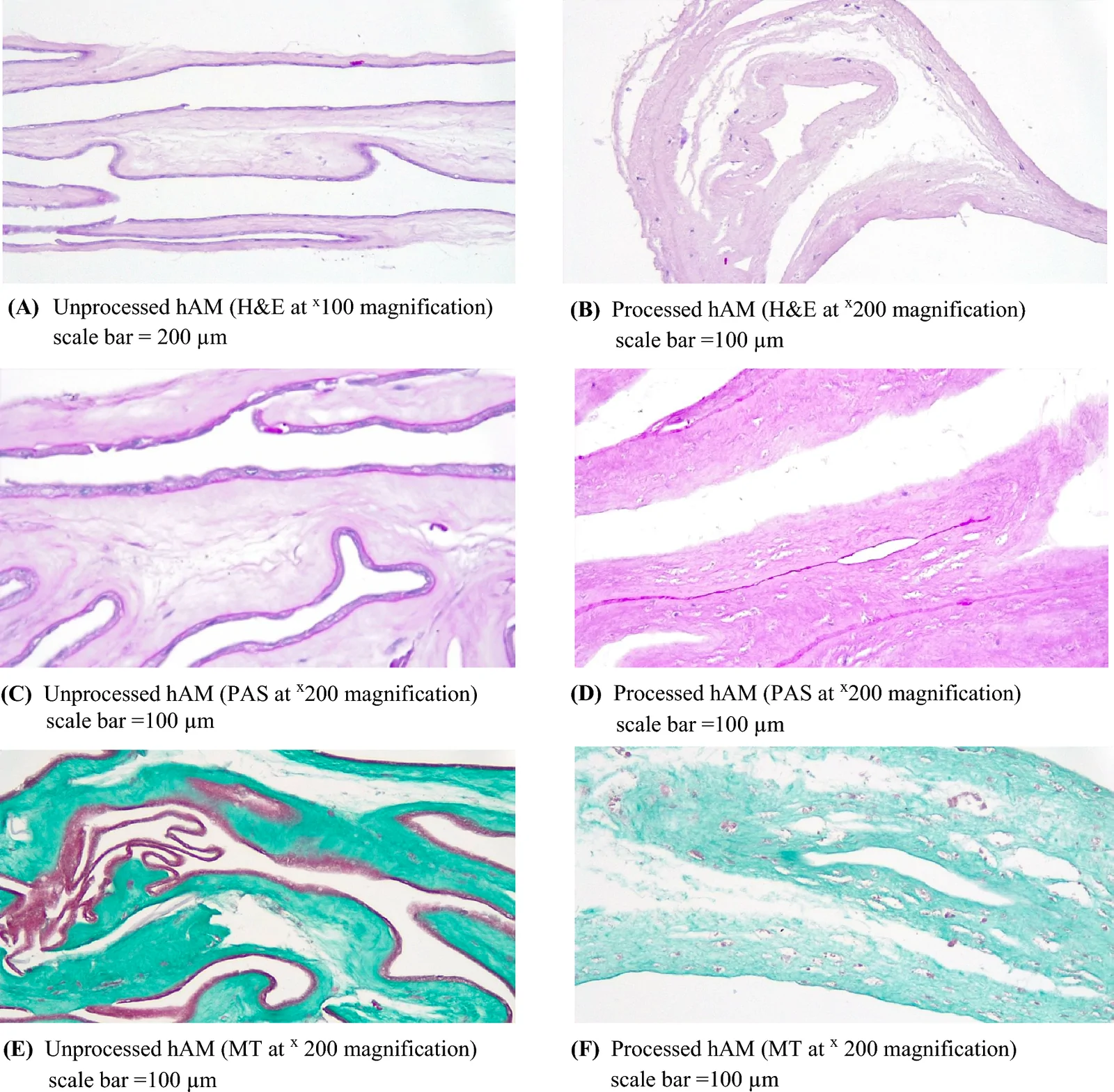
Histological evaluation of unprocessed and glycerol processed hAM

Representative micrographs (Fig. 4) demonstrated consistent structural differences between unprocessed and glycerol-processed hAM across the three staining modalities. Haematoxylin and eosin (H&E) staining showed an intact, epithelial layer with preserved cellular morphology in unprocessed samples (A), with complete epithelial loss and reduced cellularity following processing (B). Periodic acid Schiff (PAS) staining (C-D) showed a continuous basement membrane in both unprocessed (C) and processed (D) samples. Masson’s trichrome staining (E–F) highlights stromal architecture, with well-defined collagen structure in unprocessed samples and reduced collagen definition and matrix alteration following processing (F).

## Discussion

This study evaluated the effects of glycerol processing on the structural, biochemical, optical and antimicrobial characteristics of hAM following 12 months of preservation at 1–4 °C. Comparison of processed and unprocessed membranes demonstrated processing-associated changes across the evaluated parameters, while inhibition of the tested bacterial species was observed following standardized saline rinsing of the processed membranes. Collectively, these findings characterize the effects of the glycerol-based processing method under the experimental conditions of this in vitro study, without establishing clinical efficacy.

### Mechanical properties

hAM processing resulted in small but statistically significant reductions in tensile strength and tensile stress, with small effect sizes, while maximum tensile strain was not significantly altered. The 7.6% reduction in UTS observed in the present study was lower than the preservation dependent differences reported by von Versen-Hoeynck et al. (2008) who reported an approximately 46% lower tensile strength in PES/GLYC compared with air-dried membranes (von Versen-Hoeynck et al. 2008). Other processing and modification approaches can produce more substantial changes in the mechanical properties of hAM; for example, chemical cross-linking has been shown to markedly modify tensile properties (Tanaka et al. 2012). Taken together, the present findings indicate that the glycerol-processing protocol produced relatively limited changes in the measured tensile properties of hAM under the experimental conditions used. However, the clinical significance of these changes, including their potential effects on membrane handling, wound conformity, and mechanical performance during burn wound application, cannot be determined from in vitro tensile testing alone.

### Optical transmittance and thickness

The increased optical transmittance indicates that processed hAM permitted a greater proportion of incident light to pass through the membrane under the measurement conditions. Although this corresponds to increased optical transparency, which is an important functional characteristic in ophthalmic applications (Walkden 2020), it is not a primary functional requirement for burn wound coverage. Optical transparency may offer a secondary practical advantage by allowing visual inspection of the wound bed without removing the dressing. This could facilitate monitoring of re-epithelialization and visible changes suggestive of infection or wound deterioration (Rashaan et al. 2017). Glycerol-induced optical clearing has been described in biological tissues and is attributed to mechanisms including tissue dehydration, refractive-index matching, and reduced light scattering (Tuchin 2005). In this study, the increased transmittance should be interpreted alongside the histological findings demonstrating epithelial and stromal cellular depletion with preservation of a continuous basement membrane. The change in optical properties may therefore reflect processing-associated alterations in tissue composition, including reduced cellular content, rather than improved membrane integrity per se.

hAM processing caused a statistically significant reduction in thickness, but the absolute change of 0.001 mm was minimal and remained within the clinically acceptable range of 0.02–0.05 mm typically reported for amniotic membranes (Leal‐Marin et al. 2021).

### Antimicrobial activity

Processed hAM exhibited measurable inhibition against all five tested bacterial species after 12 months of refrigerated storage, with inhibition zone diameter varying according to bacterial species and culture medium. In contrast, unprocessed hAM stored for the same period under the same refrigerated conditions produced substantially smaller inhibition zones when tested under the same organism-specific culture conditions. Previous in vitro studies have similarly reported antibacterial effects associated with hAM against clinically relevant gram positive and gram negative bacteria including *S. aureus, E. coli,* and *P. aeruginosa* (Talmi et al. 1991; Mao et al. 2017). In addition to measurable inhibition zones, no bacterial growth was observed directly beneath the hAM discs. Similar findings have been reported by Talmi et al. (1991), who observed inhibition of bacterial growth beneath amniotic membrane despite the absence of surrounding inhibition zones, suggesting that direct membrane contact or barrier effects may contribute to the observed antimicrobial response.

The substantially larger inhibition-zone diameters observed in glycerol-processed compared with unprocessed hAM should, however, be interpreted cautiously. Both groups were stored for 12 months at 1–4 °C and evaluated under the same organism-specific culture conditions, with unprocessed hAM maintained in sterile saline and processed hAM preserved in glycerol. Although glycerol-processed hAM samples were thoroughly rinsed with sterile saline before antimicrobial testing, residual glycerol was not quantitatively assessed. Consequently, the greater inhibition observed following processing cannot be attributed exclusively to intrinsic antimicrobial properties of hAM, a contribution from residual glycerol cannot be excluded. The comparison therefore demonstrates a processing-associated difference in bacterial inhibition under the experimental conditions used, but does not establish the mechanism responsible for this difference.

The biological relevance of the observed inhibition in vivo also remains uncertain, as an agar-based assay does not reproduce the complex microbial and host environment of a burn wound. Furthermore, antimicrobial effects associated with amniotic membrane may not depend solely on diffusible activity, as inhibition occurring through direct membrane contact or barrier effects may not be fully represented by inhibition-zone diameter (Talmi et al. 1991). The present findings should therefore be interpreted as evidence of bacterial inhibition under the experimental conditions used rather than evidence of clinical antimicrobial efficacy.

Differences in inhibition-zone diameter were also observed when glycerol-processed hAM was evaluated on organism-specific media and Nutrient Agar, with generally larger zones observed on Nutrient Agar. These differences indicate that inhibition-zone measurements were dependent, at least in part, on the culture conditions used in the assay and highlight the importance of culture medium when interpreting agar-based inhibition measurements. The Nutrient Agar experiment was conducted only with glycerol-processed hAM and therefore provides information on variation in measured inhibition across culture conditions. These findings do not establish the mechanism or source of the observed inhibitory effect. Variation in inhibition-zone diameter was also observed among individual hAM samples.

### Histological evaluation

Histological analysis demonstrated processing-associated morphological changes in hAM, characterized by loss of the amniotic epithelial layer, reduced stromal cellularity, and altered stromal appearance. Despite these changes, PAS staining showed that the basement membrane remained visibly continuous following glycerol processing. Masson's trichrome staining demonstrated reduced collagen staining definition in processed samples, indicating alteration of the stromal matrix rather than complete preservation of native tissue architecture.

Processing-associated changes in epithelial and stromal cellularity have also been reported in previous histological evaluations of preserved hAM (von Versen-Hoeynck et al. 2008). The retention of a visibly continuous basement membrane may be relevant because the amniotic basement membrane contains extracellular matrix components involved in epithelial cell adhesion and differentiation (Niknejad et al. 2008; Mamede et al. 2012). However, the present histological findings demonstrate morphological retention only and do not establish preservation of the biological activity or functional properties of these matrix components.

The histological assessment was qualitative and intended to characterize morphological changes associated with glycerol processing. Although differences in epithelial and stromal cellularity, basement membrane continuity, and collagen staining were observed, these features were not quantitatively assessed. The findings should therefore be interpreted as descriptive evidence of processing-associated morphological changes rather than quantitative evidence of structural preservation. Future studies incorporating quantitative histomorphometry or image-based analysis of parameters such as cell density, basement membrane thickness, and collagen staining would provide a more objective assessment of these structural changes.

## Limitations

This study characterized the effects of glycerol processing and preservation on selected biomechanical, optical, antimicrobial, and histological properties of hAM. However, several limitations must be acknowledged. The study was conducted entirely in vitro, and the findings therefore cannot establish clinical efficacy or predict outcomes such as wound healing, biocompatibility, or host responses following application to burn wounds. A clinical study in patients with partial-thickness burns is currently underway to evaluate the in vivo performance and clinical efficacy of hAM processed using this protocol.

While tensile testing was conducted using ASTM D882–12 as a reference, the absence of standardized protocols specifically developed for biomechanical testing of hAM may limit reproducibility across laboratories. Tensile testing was performed following standardized overnight air-drying to minimize specimen slippage during clamping. As dehydration can alter the biomechanical behaviour of soft tissues, the measured values should be interpreted as comparative mechanical properties under standardized testing conditions rather than as absolute properties of hAM in its native hydrated state.

All processed hAM samples in the present study were evaluated following 12 months of glycerol preservation at 1–4 °C. At this time point, the membranes retained measurable biomechanical properties and produced measurable bacterial inhibition, while demonstrating the processing-associated optical and histological changes described in this study. Storage periods beyond 12 months were not evaluated; therefore, these findings should not be extrapolated beyond the assessed storage period. Further longitudinal assessment is required to determine whether these characteristics remain consistent with extended preservation.

The antimicrobial findings are also subject to methodological limitations. Although glycerol-processed hAM was thoroughly rinsed with sterile 0.9% saline before antimicrobial testing and unprocessed hAM stored under the same refrigerated conditions was included as a comparator, residual glycerol was not quantitatively assessed. A contribution of residual glycerol to the greater bacterial inhibition observed in processed samples therefore cannot be excluded, and the assay does not establish the mechanism responsible for the observed processing-associated difference. Furthermore, unprocessed hAM was evaluated only on the organism-specific media and not on Nutrient Agar; consequently, the Nutrient Agar experiment permits assessment of culture-medium-associated variation within processed hAM.

Histological evaluation was qualitative and did not include quantitative histomorphometry, biochemical analysis, or ultrastructural assessment. Although differences in epithelial and stromal cellularity, basement membrane continuity, and collagen staining were observed, these features were not quantitatively assessed. The findings should therefore be interpreted as descriptive evidence of processing-associated morphological changes rather than quantitative evidence of structural preservation. Future studies incorporating quantitative histomorphometry, image-based analysis, biochemical characterization, or ultrastructural assessment would provide a more objective evaluation of these changes.

An additional limitation is the absence of a direct comparison with other established hAM preservation methods. The paired design allowed processing-associated changes to be assessed by comparing glycerol-processed hAM with matched unprocessed tissue from the same donor, thereby reducing inter-donor variability. However, these findings do not establish the relative performance of this protocol compared with cryopreservation, lyophilization, or alternative glycerol-based preservation methods. Future studies incorporating direct preservation-method comparators are required to determine the relative effects of these approaches on relevant membrane characteristics.

## Conclusion

Following 12 months of glycerol preservation at 1–4 °C, processed hAM demonstrated measurable biomechanical properties and demonstrated bacterial inhibition, increased optical transmittance, and processing-associated histological changes. Modest reductions in tensile strength and thickness were observed, while maximum tensile strain was not significantly altered. Qualitative histological assessment demonstrated reduced cellularity and stromal modification, while the basement membrane remained visibly continuous. Collectively, these findings characterize the effects of the glycerol-based processing and preservation method on selected properties of hAM under in vitro conditions. However, the clinical significance of these findings and their implications for burn wound performance cannot be established from the present study. Further in vivo evaluation is required to determine whether these laboratory findings translate into safe and effective clinical performance.

## Data Availability

The data that support the findings of this study are available from the corresponding author upon reasonable request.
